# Integrating Molt Migration Into Physiological Assessments of Inter‐Population Stress in a Globally Endangered Species

**DOI:** 10.1002/ece3.73179

**Published:** 2026-03-02

**Authors:** Shurui Bai, Diana Solovyeva, Xiaorui Wang, Fucheng Xu, Peishan Li, Lin Wang, Bo Liu, Haitao Wang, Ye Gong

**Affiliations:** ^1^ School of Life Sciences Northeast Normal University Changchun China; ^2^ Institute of Biological Problems of the North FEB RAS Magadan Russia; ^3^ Northeast Institute of Geography and Agroecology Chinese Academy of Sciences Changchun China

**Keywords:** breeding habitat, endangered species, feather corticosterone, *Mergus squamatus*, molt migration

## Abstract

Feather corticosterone (fCORT) acts as a reliable indicator of avian physiological responses to environmental stressors. However, variation in fCORT across taxa and ecological contexts, combined with limited knowledge of the stress physiology of molt migrants (individuals moving from breeding to specific molting grounds) has left population‐level stress patterns in many species poorly understood. We compared fCORT concentrations in the globally endangered Scaly‐sided Merganser ( *Mergus squamatus* , SSME) between its two remaining breeding populations exposed to different stress regimes, while accounting for molting habitat variation inferred from stable isotope analyses. By controlling for the generally higher fCORT concentrations in molt migrants, population‐level variation was primarily driven by habitat‐specific stressors. Elevated fCORT levels in the Russian population are indicative of heightened physiological stress, whereas the comparatively low fCORT levels observed in the Chinese population may reflect habituation to chronic disturbance. Meanwhile, the divergence in fCORT between populations may be further mediated by population‐specific factors, such as migration‐related energetic demands and habitat conditions. These contrasting stress profiles highlight the importance of adopting population‐specific approaches in conservation planning. Our findings reinforce the growing recognition that physiological indicators offer a powerful framework for assessing population‐level responses to differing habitat conditions. Moreover, given the widespread occurrence of heterogeneous molt‐movement strategies among birds, integrating molt‐related movement ecology into conservation physiology can improve our ability to identify vulnerable subpopulations, refine stress‐based monitoring tools, and design management actions that better accommodate intra‐specific diversity across avian taxa.

## Graphical abstract

Feather corticosterone (fCORT) provides a powerful indicator of population‐level physiological responses to human disturbance, yet its interpretation is complicated by variation across taxa, habitats, and molt‐movement strategies. Using stable isotope analyses to account for molt migrants in the globally endangered Scaly‐sided Merganser ( *Mergus squamatus* ), we show that elevated fCORT in the Russian breeding population reflects heightened stress, whereas markedly lower levels in the Chinese population likely indicate habituation to chronic disturbance. These contrasting stress profiles highlight the importance of integrating molt‐related movement ecology into conservation physiology to identify vulnerable subpopulations, refine stress‐based monitoring tools, and design population‐specific management actions.

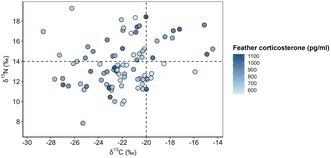

## 1 Introduction

Monitoring variation in stress physiology can improve assessments of population status in conservation‐challenged species (Madliger et al. 2021), because stress responses can indicate fitness consequences of environmental stressors before detectable population declines, particularly in endangered species (e.g., Kilgour et al. 2024). In vertebrates, the hypothalamic—pituitary—adrenal (HPA) axis regulates stress responses through glucocorticoid (GC) release, with corticosterone serving as the primary GC in birds as well as other vertebrates (Romero 2004). Acute GC elevations promote survival by reallocating energy to essential functions and restoring homeostasis, whereas chronic elevations can impair immunity, alter behavior, and reduce reproductive success (Pérez‐Ortega and Hendry 2023). GCs have thus become widely used biomarkers linking physiology to demography and guiding conservation management, revealing how environmental conditions and anthropogenic disturbances affect free—ranging wild animals (e.g., Belanger et al. 2016; Messina et al. 2020; Goff et al. 2020; Scheun et al. 2021; Kachamakova et al. 2021; French et al. 2022; Kilgour et al. 2024). However, GC responses vary across taxa and contexts: while some species and/or populations show elevated levels under disturbances (Funes et al. 2015; McLennan et al. 2019; Grunst et al. 2020), others exhibit habituation or even suppressed secretion with prolonged exposure (Rimbach et al. 2013; Nelson et al. 2015; Scheun et al. 2021).

Keratinized tissues have become valuable indicators of chronic stress, as circulating corticosterone (CORT) is continuously deposited during tissue growth, providing an integrated record of hormone levels over time (Bortolotti et al. 2008). Among these tissues, feathers are particularly useful in birds, offering an accessible and noninvasive means of assessing physiological stress (Fairhurst et al. 2013), compared with baleen in whales (Hunt et al. 2014), claws in turtles (Baxter‐Gilbert et al. 2014), and scutes in alligators (Hamilton et al. 2018). Feather corticosterone (fCORT) reflects both acute and chronic responses, yielding insight into how individuals and populations respond to past stress during feather growth (Jenni‐Eiermann et al. 2015; Kilgour et al. 2024). In contrast, traditional matrices such as blood, urine, and feces capture only short‐term fluctuations and are often confounded by capture‐induced stress or logistical challenges (Romero and Reed 2005; Creel 2001; Cabezas et al. 2007). Consequently, fCORT has emerged as a robust biomarker for assessing long‐term physiological effects of habitat conversion, degradation, and other anthropogenic pressures, providing critical insights for wildlife conservation and management (Fairhurst et al. 2013; Pérez‐Ortega and Hendry 2023).

Many bird species remain on or near their breeding grounds to molt (Salomonsen 1968; Kjellén 1994; Pyle et al. 2018), potentially allowing fCORT to reflect hormonal exposure to conditions within the breeding habitat range. However, when local ecological conditions are limited, some individuals move to more favorable sites—a behavior known as molt migration (Salomonsen 1968). This strategy, common in wildfowl and passerines (Salomonsen 1968; Jehl 1990; Pyle et al. 2018), likely provides energetic benefits (Jenni and Winkler 2020), as food‐rich habitats can promote feather growth and mitigate nutritional stress (Kjellén 1994; Will et al. 2014; Pageau et al. 2020). Yet, molt migrants may also show elevated activity and metabolic demands during molt (Vīgants et al. 2023; Crossin et al. 2012), suggesting that stress levels are not solely governed by food availability (Bourgeon et al. 2014). Understanding the stress physiology of molt migrants is therefore crucial, both for their effective conservation and for accurately interpreting fCORT variation among populations, although empirical evidence remains scarce for most migratory species.

In this study, we used fCORT as a physiological biomarker to assess the population status of the globally endangered Scaly‐sided Merganser ( *Mergus squamatus* , SSME), which has a highly restricted range and is listed as Endangered by the IUCN (BirdLife International 2025). Historically, the species bred across temperate riparian old‐growth forests of Southeast Russia and Northeast China (BirdLife International 2025), but recent field surveys and habitat models indicate that its breeding range has contracted to two main regions: the Sikhote‐Alin Range in Russia and the Changbai Mountains in China (Solovyeva, Liu, et al. 2014; Xu, Solovyeva, et al. 2021). These populations experience contrasting threats—the Russian population (RUS), though larger, faces localized pressures such as poaching and fishing‐net entanglement, whereas the smaller Chinese population (CHN) is exposed to chronic and widespread stressors, including dam construction, water pollution, subsistence farming, and increasing disturbance from urban expansion and tourism (BirdLife International 2025; Solovyeva et al. 2017). Such divergent pressures likely impose differing physiological burdens, underscoring the need for comparative assessments of stress hormone levels as early indicators of sublethal effects.

Moreover, evidence indicates that this species undertakes molt migration, with some individuals molting in freshwater rivers and others—particularly failed breeders—dispersing to nonfreshwater (i.e., brackish or marine) habitats (Solovyeva, Newton, et al. 2014; Solovyeva et al. 2016). These contrasting environments, potentially differing in ecological and environmental conditions, such as food availability, competition and salinity, may drive intra‐population variation in stress physiology (Will et al. 2014; Reese et al. 2020; Tornabene et al. 2021). Stable isotope (SI) analysis has proven effective in distinguishing freshwater from nonfreshwater molting sites within the SSME (Solovyeva, Newton, et al. 2014; Solovyeva et al. 2016), providing a valuable complement to fCORT‐based assessments. Accordingly, beyond comparing fCORT concentrations between the RUS and CHN populations, our study also accounts for potential variation in hormone levels associated with molting habitats identified through SI analysis, thereby offering a more integrated understanding of physiological stress in this endangered species.

## 2 Materials and Methods

### 2.1 Sample Collection

**FIGURE 1:**
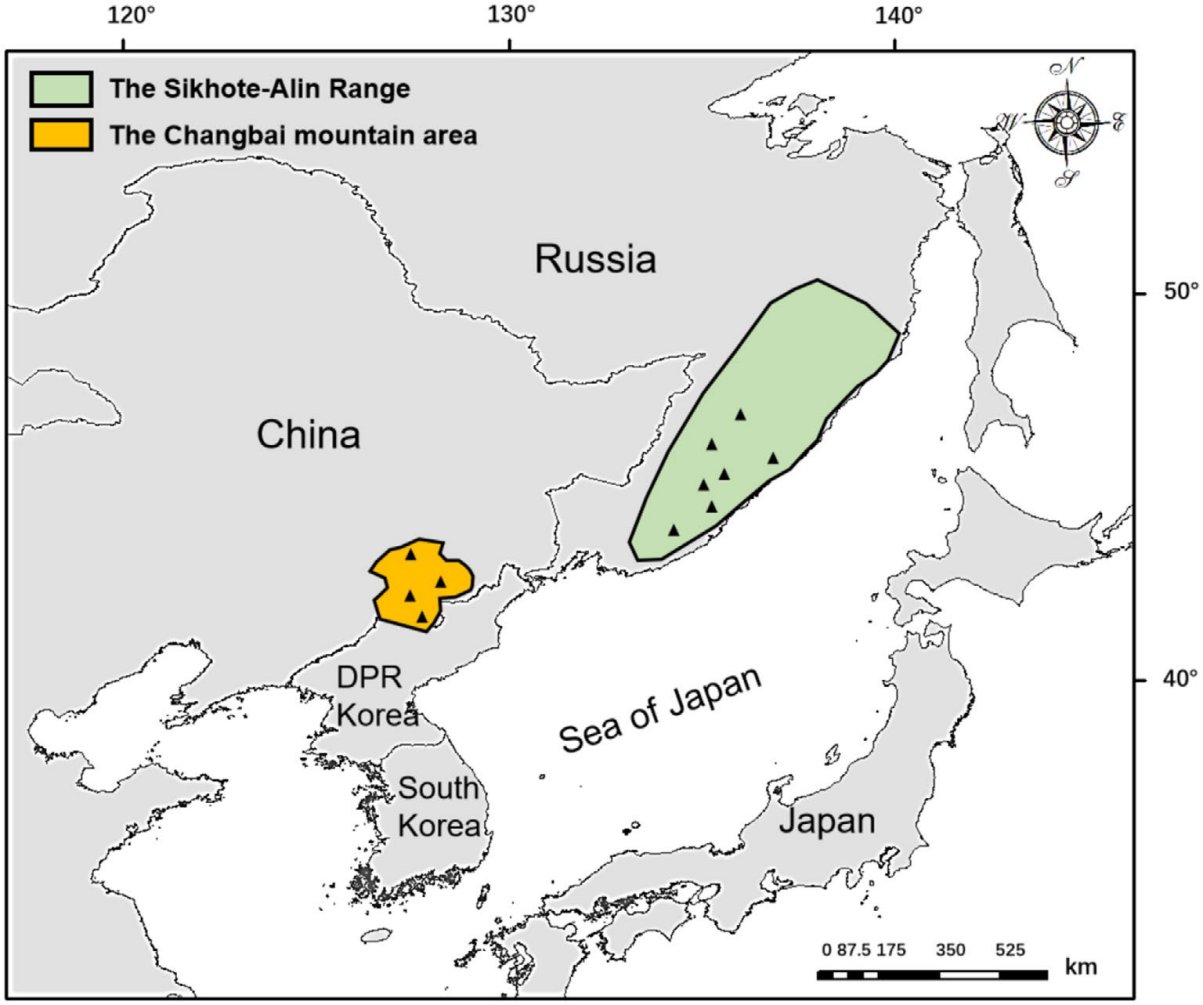
Map of the main breeding areas (i.e., the Sikhote‐Alin Range of southeast Russia and the Changbai Mountain area of Notheast China) of the scaly‐sided merganser ( *Mergus squamatus* ) and fCORT sampling locations (represented by triangles).

Feather samples were obtained from long‐term demographic monitoring sites distributed across the core breeding grounds in the Sikhote‐Alin Range and the Changbai Mountains (Figure 1; Solovyeva, Liu, et al. 2014; Xu, Solovyeva, et al. 2021). We collected nest lining feathers from nesting material after fledging (*n* = 80) from both artificial nest boxes and natural tree cavities, supplemented with samples from naturally deceased individuals (*n* = 3) and specimens archived at the Museum of the School of Life Sciences, Northeast Normal University (*n* = 2), Changchun, China. Given the potential for low repeatability in feather hormone measurements (Harris et al. 2016), we reduced this source of variability by consistently sampling feathers from the same body region. All samples were stored in envelopes at −4°C until analysis. Consistent with patterns reported for other duck species (e.g., Kilgour et al. 2024), female Scaly‐sided Mergansers typically undergo molt between June and August (Solovyeva et al. 2016). Therefore, the fCORT measured in these feathers should reflect physiological stress and environmental conditions experienced during approximately one month within a comparable seasonal window across all samples.

### 2.2 Hormone Assay

Feathers were visually inspected for external urate contamination and, if present, removed using water and gentle wiping. Sample preparation followed established protocols (Bortolotti et al. 2008; Lattin et al. 2011), with feather mass standardized to ≥ 20 mg to minimize nonlinear effects of sample size on hormone concentration (Romero and Fairhurst 2016).

Each feather section was cut into fragments of less than 5 mm using stainless‐steel scissors and extracted overnight in 10 mL of methanol at 50°C under constant agitation. The methanol extract was then separated from feather material by vacuum filtration through 47 mm glass microfiber filters. Following filtration, the extraction vial, filter, funnel, and collection flask were rinsed with approximately 3 mL of methanol, and the rinses were combined with the filtrate. Extracts were evaporated to dryness under vacuum and stored at −20°C until analysis.

Corticosterone concentrations were quantified using a commercially available enzyme‐linked immunosorbent assay (ELISA) kit (Cat. No. ADI‐900‐097, Enzo Life Sciences), which has been successfully applied in previous feather corticosterone studies (Kouwenberg et al. 2015; Glucs et al. 2018). Assays were conducted according to the manufacturer's instructions, with samples re‐suspended and diluted in the kit's assay buffers before measurement.

### 2.3 Stable Isotopes Analysis

Feather samples were cleaned to remove surface contaminants, rinsed with distilled water, dried at 50°C to constant weight, and then ground into a fine, homogeneous powder. Approximately 0.15–0.25 mg of powdered feather material was weighed into pre‐cleaned tin capsules and sealed.

Samples were combusted in a Flash HT2000 elemental analyzer (Thermo Fisher Scientific, USA) at 980°C under an oxygen‐enriched atmosphere, converting them into CO_2_ and N_2_ gases. The gases were carried by helium (100 mL/min) through a molecular sieve GC column (65°C) and introduced into a MAT253 isotope ratio mass spectrometer via a Conflo IV interface for isotopic measurement.

Isotope ratios were expressed in δ notation (‰) relative to international standards: Vienna Pee Dee Belemnite (V‐PDB) for δ^13^C and atmospheric nitrogen (AIR) for δ^15^N. Laboratory standards (USGS40, USGS41a, USGS42) were used for calibration and quality control, with USGS42 inserted every 12 samples. Analytical precision was typically ±0.1‰ for δ^13^C and ±0.2‰ for δ^15^N.

### 2.4 Identifying Nonfreshwater Molt Migration

To identify individuals undertaking nonfreshwater molting migration, feather δ^13^C and δ^15^N values were analyzed as ecological indicators of habitat type. Following established isotopic thresholds, feathers with δ^13^C values below −20‰ were considered to reflect freshwater environments, whereas δ^15^N values above 14‰ indicated nonfreshwater (i.e., brackish or marine) foraging (Solovyeva, Newton, et al. 2014). By integrating these criteria, individuals were classified as molting in either freshwater or nonfreshwater environments based on the isotopic signatures recorded in their feathers.

### 2.5 Statistical Analyses

A General Linear Model (GLM) was used to analyze feather corticosterone (fCORT) concentrations, with breeding habitat (Russia vs. China) and molting habitat (freshwater vs. nonfreshwater) included as fixed factors. Although the majority of samples from both populations were collected after 2000, samples from the China population span 2003–2024, whereas samples from the Russia population were primarily collected during 2005–2023, with two additional museum specimens collected in 1950. Sampling year was therefore included as a covariate in the analyses to account for potential temporal variation in physiological stress associated with long‐term change (Kilgour et al. 2024). Partial *R* ^2^ values were calculated using the *rsq* package to quantify the relative contribution of each explanatory variable to variation in fCORT concentrations. To meet the assumption of normality, fCORT data were log‐transformed prior to analysis. The distribution of residuals was assessed using the Kolmogorov–Smirnov test (*D* = 0.11, *p* = 0.23). All statistical analyses were conducted in R version 4.5.0 (R Core Team 2025) within RStudio version 2024.12.1 + 563.

## 3 Results

**FIGURE 2:**
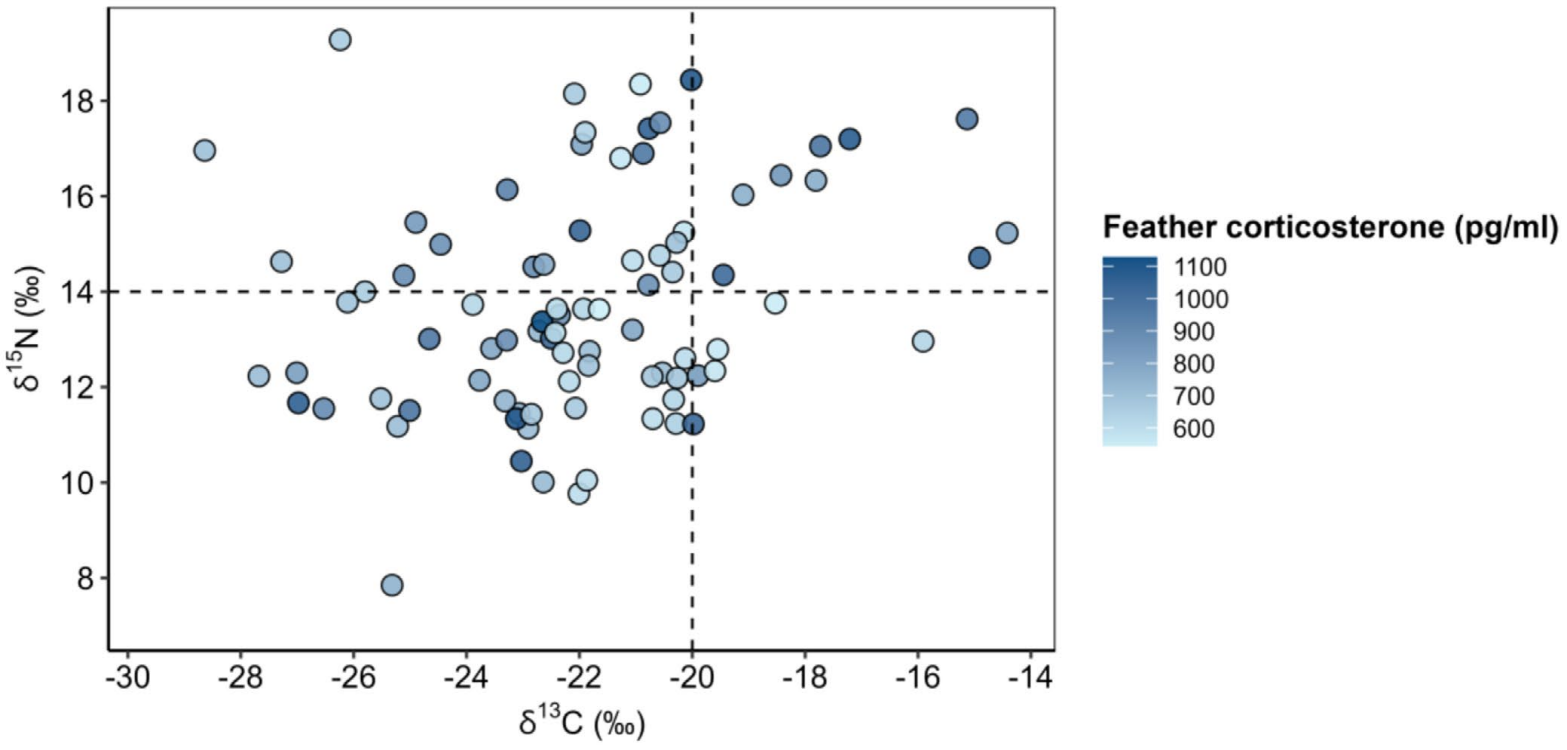
Stable isotope values (δ^13^C and δ^15^N) from feathers of Scaly‐sided Mergansers sampled in southeastern Russia and northeastern China. The vertical dashed line at −20‰ (δ^13^C) and the horizontal dashed line at 14‰ (δ^15^N) indicate the cutoff thresholds distinguishing freshwater from nonfreshwater (i.e., brackish or marine) signatures (Solovyeva, Newton, et al. 2014).

**FIGURE 3:**
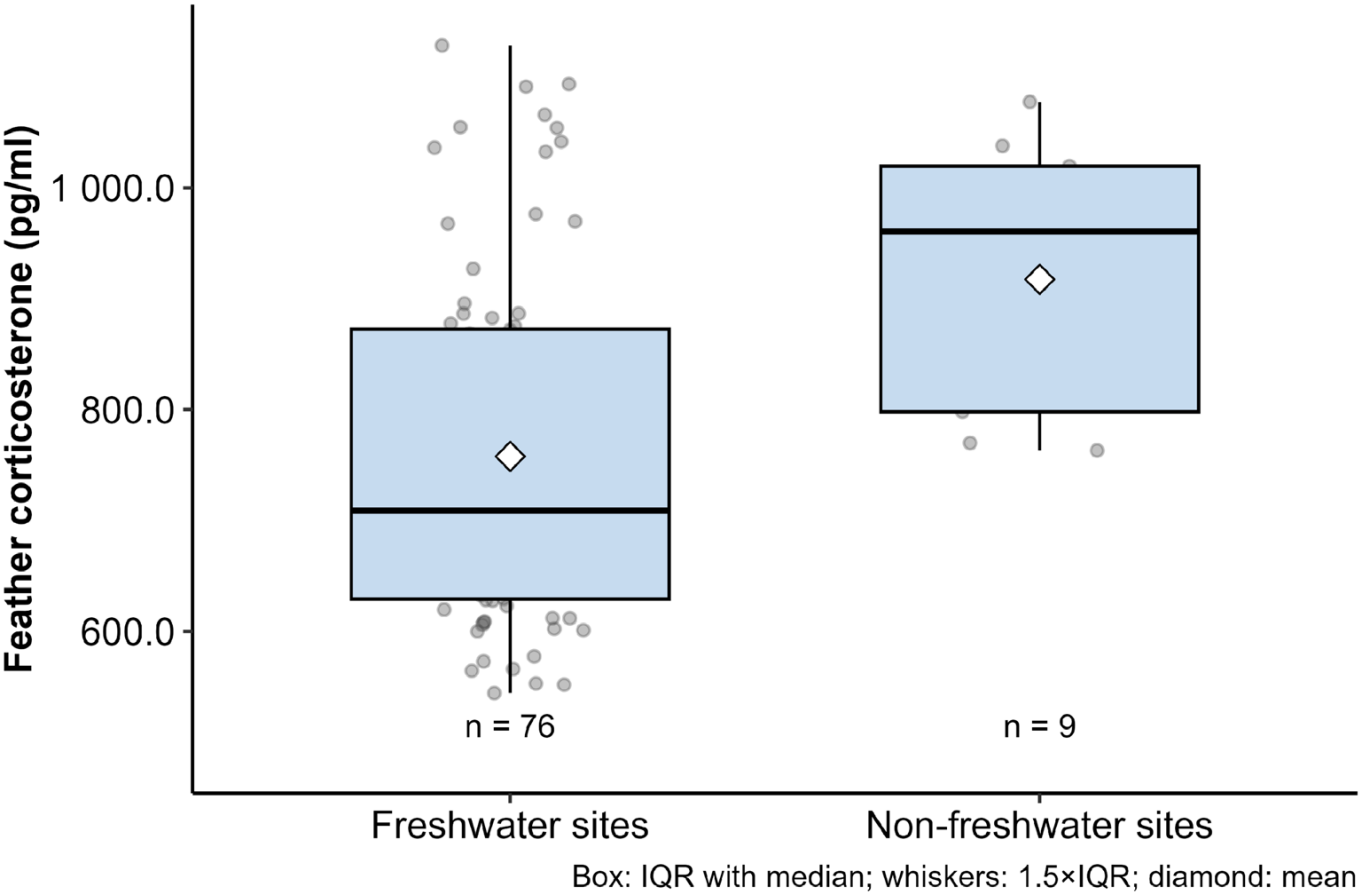
Comparison of feather corticosterone (fCORT) concentrations in Scaly‐sided Mergansers molting at freshwater versus nonfreshwater (i.e., brackish or marine) sites.

**FIGURE 4:**
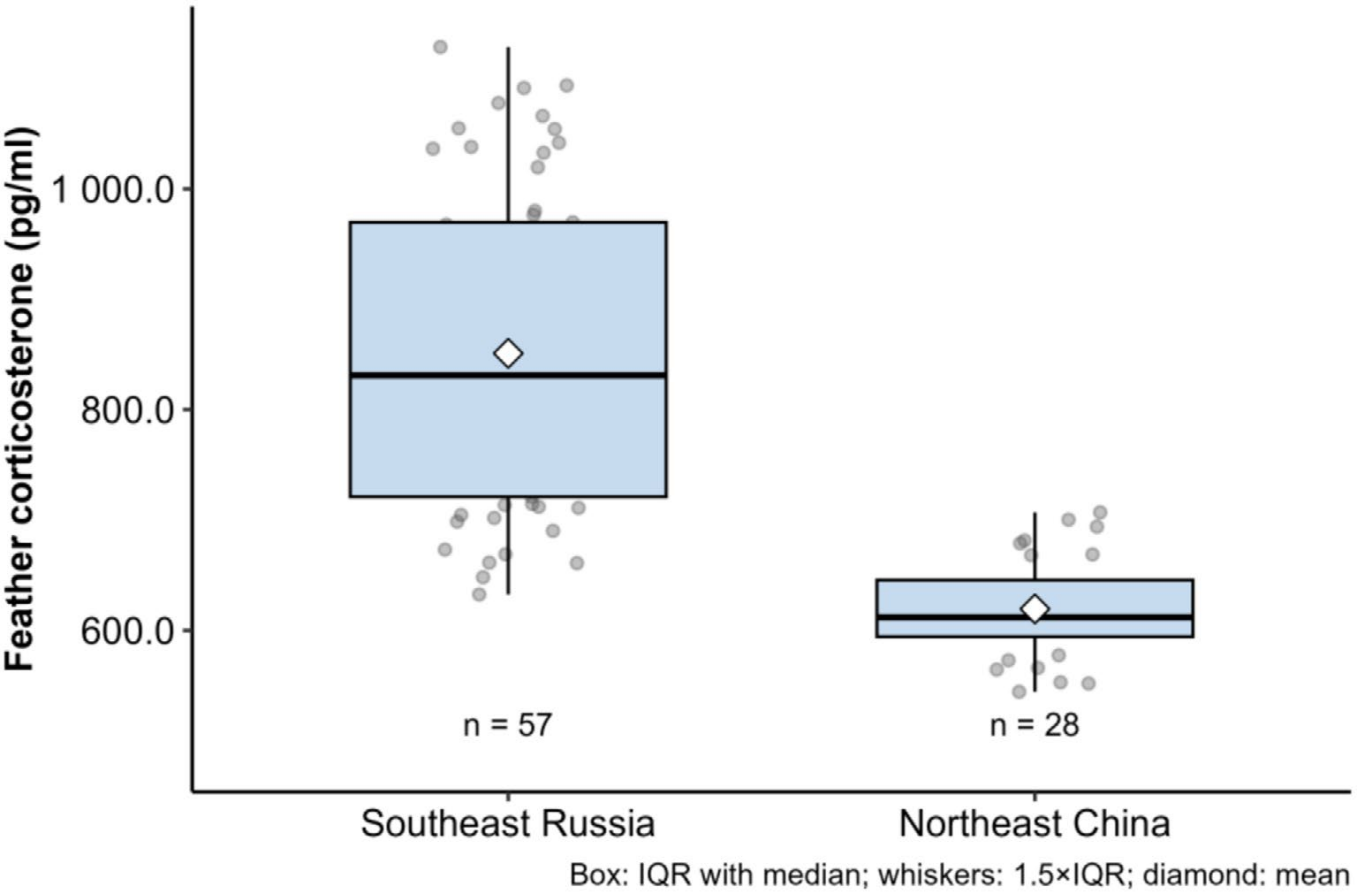
Comparison of feather corticosterone (fCORT) concentrations in Scaly‐sided Mergansers originated from Southeast Russia and Northeast China.

Based on δ^13^C and δ^15^N values, 89.4% (*n* = 76) of the sampled individuals were identified as molting in freshwater environments, while approximately 10% (*n* = 9) molted in nonfreshwater habitats (Figure 2). Notably, all individuals undergoing nonfreshwater molting originated from the Russian breeding population. GLM analysis revealed that the mean fCORT concentration of nonfreshwater molting individuals (917.50 ± 122.81 pg. mL^−1^) tended to be higher than that of freshwater molting individuals (757.18 ± 157.27 pg mL^−1^; *p* = 0.06, partial *R* ^2^ = 0.04; Figure 3). However, fCORT concentrations were primarily explained by breeding habitat (*p* < 0.01, partial *R* ^2^ = 0.48), with significantly higher mean values observed in Russian samples (850.93 ± 141.20 pg mL^−1^) compared to those from China (619.49 ± 46.58 pg mL^−1^; Figure 4). Additionally, fCORT concentrations did not differ significantly among years (*p* = 0.94).

## 4 Discussion

This study provides the first evidence of inter‐population variation in feather corticosterone (fCORT) among breeding populations while accounting for the influence of molt migration. Although nonfreshwater molt migrants exhibited higher fCORT concentrations, population‐level variation was primarily driven by habitat‐related chronic stressors, suggesting that breeding habitat conditions—rather than molt migration—play a dominant role in shaping fCORT patterns in this endangered species.

Heterogeneous molt–movement responses are widespread among birds (Salomonsen 1968; Jehl 1990; Pyle et al. 2018) and likely contribute to individual variation in stress physiology, yet they are rarely considered when interpreting inter‐population physiological differences (but see Bourgeon et al. 2014). Molt migrants, including those reported in the SSME (Solovyeva, Newton, et al. 2014; Solovyeva et al. 2016), are often younger individuals or failed breeders originating from resource‐poor territories that disperse to alternative molting sites (Rohwer et al. 2005; Pyle et al. 2018). Such dispersal may help alleviate ecological pressures in breeding rivers, including intra‐specific competition with successful brood‐rearing females, food limitation, and predation risk, particularly during the energetically demanding flightless molt period (Clinchy et al. 2004; Reese et al. 2020; Noreikienė et al. 2021). Moreover, because failed breeders generally exhibit lower baseline corticosterone (CORT) levels than successful breeders (Crossin et al. 2013), they may be better able to cope with the physiological costs associated with dispersal to nonfreshwater habitats.

Contrary to expectations, SSME individuals molting in the nonfreshwater environment exhibited elevated fCORT levels. As molting is energetically demanding, these elevated levels likely reflect increased energy expenditure rather than stress reduction. Molt migration may allow individuals to exploit food‐rich habitats outside their breeding territories, thereby ensuring feather quality and the completion of molting (Kjellén 1994). Supporting this interpretation, previous studies have reported increased foraging effort and activity during molt, behaviors known to elevate CORT levels (Wingfield et al. 1983; Vīgants et al. 2023). Furthermore, unlike other true seaducks, such as eiders, SSME lacks the ability to extract salt and shows marine isotopic signatures in its feathers (Solovyeva, Newton, et al. 2014). The hypersaline environments used by nonfreshwater molt migrants likely impose osmotic and energetic challenges, elevating metabolic demands and stress hormone secretion (Gutiérrez et al. 2011, 2012; Tornabene et al. 2021). At the same time, marine and estuarine habitats may provide richer prey resources and alternative ecological conditions compared with freshwater systems. Thus, the use of nonfreshwater molting habitats may represent an adaptive trade‐off, whereby individuals tolerate elevated physiological stress in exchange for improved foraging opportunities and reduced ecological constraints, particularly when breeding has failed. Although molt migrants constitute only a small portion of the SSME population, their unique physiological responses underscore the need to consider these individuals in future monitoring and management efforts aimed at ensuring the long‐term resilience of the species.

Beyond molt migration, differences in fCORT between the two remaining SSME breeding populations likely reflect variation in human disturbance (Pérez‐Ortega and Hendry 2023). Anthropogenic activities can disrupt foraging, resting, and molting, elevating glucocorticoid (GC) levels in free‐ranging birds (Tarjuelo et al. 2015). For instance, little bustards ( *Tetrax tetrax* ) showed higher vigilance and GC levels during hunting weekends but lower GC levels when undisturbed (Tarjuelo et al. 2015). Similarly, poaching remains a serious threat to SSMEs in southeast Russia (BirdLife International 2025) and may contribute to higher GC levels there. However, because poaching occurs mainly over a short period (~2 weeks) in early spring (Solovyeva et al. 2017), it is unlikely to influence feather CORT deposition during molt. Moreover, relative to the northeast China population, whose habitat experiences more pervasive human influence such as conversion and degradation (Pérez‐Ortega and Hendry 2023), the southeast Russia population occupies extensive suitable forest–river habitats (Xu, Solovyeva, et al. 2021), which may buffer individuals from chronic stress. In contrast, other anthropogenic threats that directly overlap with the flightless molting period may still contribute to elevated stress. For example, drowning in fishing nets, a major source of mortality during June–August (Solovyeva et al. 2017), could impose sustained disturbance and physiological costs on molting individuals, potentially eliciting prolonged glucocorticoid responses.

Unexpectedly, the Northeast China population exhibited lower GC levels despite inhabiting more disturbed areas (figure S5 in Xu, Solovyeva, et al. 2021), in contrast to studies reporting lower GC concentrations in birds from undisturbed environments (e.g., Thiel et al. 2005; Ellenberg et al. 2007; Strasser and Heath 2013). These reduced levels may represent an adaptive adjustment to persistent anthropogenic stressors, achieved through decreased baseline concentrations and/or attenuated stress responsiveness, thereby minimizing the physiological costs of chronic activation of the HPA axis (Cyr and Romero 2009). With rapid regional development—for instance, the population of Songjianghe in the Changbai Mountain area is projected to increase fivefold alongside the expansion of highways and railways—SSMEs are increasingly exposed to human disturbance from traffic, tourism, agriculture, and fisheries (Xu, Gong, and Wang 2021). Repeated exposure to nonaggressive human activities can induce habituation, leading to lower baseline GC levels (Scheun et al. 2021; Ibáñez‐Álamo et al. 2020). Supporting this interpretation, individuals in Northeast China exhibited shorter flight initiation distances than those in Russia (~90 m vs. 190 m; Xu, Gong, and Wang 2021), indicating habituation to human presence. Alternatively, chronic exposure might lead to desensitization of adrenocorticotropic hormone (ACTH) responsiveness, resulting in a generalized suppression of both stress reactivity and baseline GC secretion (Cyr and Romero 2009). However, despite shorter FIDs, increased vigilance and elevated fecal CORT levels in disturbed areas (Xu, Gong, and Wang 2021) suggest that certain human‐related stimuli continue to elicit stress responses, though these responses appear insufficiently intense or sustained to trigger chronic physiological stress (Maréchal et al. 2011). Collectively, and consistent with findings in several other avian species exposed to recurrent anthropogenic disturbance (e.g., Pichegru et al. 2016; Chiew et al. 2019; Scheun et al. 2021; Ibáñez‐Álamo et al. 2020; Brodin and Watson 2023), these results indicate that the attenuation of stress responses in SSMEs is more consistent with habituation—a stimulus‐specific behavioral and physiological adjustment—than with generalized desensitization (Cyr and Romero 2009).

Although habitats in Northeast China have been substantially altered by deforestation, hydrological engineering, and settlement expansion (Solovyeva et al. 2017), recent conservation actions appear to have mitigated some of the physiological stress experienced by SSMEs. Local governments have strengthened species protection by installing artificial nest boxes, improving water quality, and enhancing patrolling and law enforcement (https://jllc.jl.gov.cn/). These actions have likely improved habitat quality and reduced chronic stress exposure, as evidenced by lower hormone levels in individuals from translocation programs and protected areas (Pérez‐Ortega and Hendry 2023; Kilgour et al. 2024), contributing to the lower baseline CORT levels observed in this population.

Variation in other ecological and environmental factors may also influence the energetic balance of individuals from the two populations. For example, the breeding and molting habitats in Russia harbor a high diversity of predators, including 15 species of mammalian and 11 avian predators potentially dangerous to SSME in the Sikhote‐Alin region (Khokhryakov and Shokhrin 2002). In contrast, predation on SSME has been only occasionally observed in the Changbai Mountain area, likely due to a “human shield” effect (Gaynor et al. 2025). Beyond predation, migration‐related factors may further contribute to endocrine differences between populations. Russian breeding and molting areas occur at higher latitudes and may require longer migratory routes, increasing energetic expenditure and exposure to harsher climatic conditions. Elevated energetic demands associated with long‐distance migration can influence CORT regulation during subsequent stages such as molt, potentially contributing to higher integrated fCORT levels (Mikkelsen et al. 2021; Lynn et al. 2022). By comparison, shorter migratory distances to Chinese breeding areas may reduce energetic costs, allowing individuals to better buffer migration‐related demands and maintain lower glucocorticoid levels. Overall, these patterns likely reflect the combined effects of migration distance, climatic context, and individual condition rather than any single stressor acting in isolation (Legagneux et al. 2013).

Our results provide preliminary evidence for inter‐population differences in fCORT, with SSME individuals breeding in Russia exhibiting higher stress indices, whereas those in China show comparatively lower values. Although fCORT measurements can show low repeatability at the individual level and should therefore be interpreted cautiously when based on single feathers (Harris et al. 2016; Romero and Fairhurst 2016), our results nonetheless highlight consistent relative differences at the population scale. Genetic evidence indicates weak but significant differentiation with high gene flow between the Russian and Chinese breeding subpopulations, suggesting they remain partially connected, likely via male‐mediated dispersal at shared wintering grounds (Shen et al. 2024). However, strong female philopatry and breeding‐site fidelity may maintain relatively stable local breeding groups (unpublished data；Shen et al. 2024), allowing the two regions to experience distinct ecological and anthropogenic conditions. This shallow but persistent structure could therefore contribute to population‐specific habituation or sensitization to different stressors in China versus Russia habitats.

Elevated CORT concentrations can impair immune function (Råberg et al. 1998), increase oxidative stress (Costantini et al. 2008), and inhibit feather growth during molt (Romero et al. 2005); thus, they warrant attention in the Russian population. These findings emphasize the need to prioritize habitat protection at key breeding and molting sites in Russia, whereas disentangling the relative contributions of ecological and anthropogenic stressors in order to identify the main drivers of elevated physiological stress and develop targeted management responses. Additionally, the occurrence of a small subset of individuals using nonfreshwater molting habitats suggests that conservation planning should account for alternative molting strategies and include coastal or estuarine environments in future monitoring efforts.

In contrast, although comparatively low fCORT levels in the Chinese population may reflect habituation to chronic disturbance, habituation does not fully mitigate the adverse effects of long‐term exposure (Pérez‐Ortega et al. 2023). Moreover, excessively low CORT levels may blunt vigilance, increasing susceptibility to real threats and potentially affecting offspring performance (Brodin and Watson 2023). This underscores the importance of maintaining long‐term stability of breeding rivers in China, while monitoring potential sublethal impacts that may not be captured by stress hormones alone. Finally, further work integrating larger sample sizes, complementary physiological metrics, and analyses of key threat factors across the annual cycle will help strengthen these population‐level inferences and improve stress‐based conservation monitoring.

Our findings reinforce the growing recognition that physiological indicators, particularly GC metrics, provide a powerful framework for assessing population‐level responses to different habitat conditions (Bergman et al. 2019). As anthropogenic pressures continue to reshape habitats and as conservation increasingly relies on translocation and ex‐situ management, many species are now exposed to spatially heterogeneous and temporally variable stressors (Kilgour et al. 2024; Luo et al. 2024). However, the diversity of species‐specific physiological responses and the heterogeneity of individual movement within populations pose significant challenges for interpreting population‐level physiological signals (Bergman et al. 2019). In the present study, the pronounced inter‐population difference in fCORT among SSME populations underscores these challenges, indicating that intra‐specific variation in endocrine profiles may reflect underlying ecological or behavioral divergence. Moreover, the observed hormonal divergence between SSME molt migrants and other conspecifics highlights the importance of incorporating molt–movement strategies when interpreting physiological variation in other species, including both waterbirds and landbirds (Salomonsen 1968; Jehl 1990; Pyle et al. 2018). More broadly, integrating molt‐related movement ecology into conservation physiology frameworks can enhance our ability to identify vulnerable subpopulations, refine stress‐based monitoring tools, and design management actions that account for intra‐specific diversity (Bergman et al. 2019).

## Author Contributions

**Shurui Bai:** formal analysis (equal), methodology (equal), resources (equal), writing – original draft (lead). **Diana Solovyeva:** conceptualization (supporting), data curation (equal), investigation (equal), methodology (supporting), resources (equal), writing – review and editing (equal). **Xiaorui Wang:** investigation (equal), methodology (equal), resources (equal), software (equal), writing – original draft (equal). **Fucheng Xu:** formal analysis (equal), investigation (equal), methodology (equal), resources (equal), software (equal). **Peishan Li:** investigation (equal), methodology (supporting). **Lin Wang:** methodology (equal), software (equal). **Bo Liu:** methodology (equal). **Haitao Wang:** project administration (equal), supervision (equal). **Ye Gong:** conceptualization (lead), data curation (lead), formal analysis (lead), funding acquisition (lead), writing – original draft (lead), writing – review and editing (lead).

## Funding

This work was financially supported by the National Natural Science Foundation of China (No. 3210127), the Natural Science Foundation of Jilin Province (No. 20230101157JC), and the project Monitoring and Ecological Assessment of Typical Animal Groups in the Liaohe Estuary Wetland (No. JH25‐211100‐00369).

## Ethics Statement

All field experiments were conducted in compliance with the relevant laws and regulations of China and Russia, where the study was carried out. The sampling procedures involved noninvasive collection methods and did not cause harm or distress to the animals.

## Conflicts of Interest

The authors declare no conflicts of interest.

## Data Availability

The datasets of the current study are available in the figshare repository at https://figshare.com/s/ac19750bde89697ac70b.
